# LncRNA SNHG3 Promotes Gastric Cancer Cells Proliferation, Migration, and Invasion by Targeting miR-326

**DOI:** 10.1155/2021/9935410

**Published:** 2021-06-28

**Authors:** Jun Rao, Jinjin Fu, Chuchen Meng, Jin Huang, Xiangrong Qin, Shaohua Zhuang

**Affiliations:** ^1^Department of Gastroenterology, Changzhou No. 2 People's Hospital, The Affiliated Hospital of Nanjing Medical University, No. 29 Xinglong District, Changzhou 213000, China; ^2^Department of Endocrinology, Changzhou No. 2 People's Hospital, The Affiliated Hospital of Nanjing Medical University, Changzhou, China

## Abstract

The function and possible mechanism of lncRNA Small Nucleolar RNA Host Gene 3 (SNHG3) in GC have not been fully studied. The aim of our study was to investigate the role of SNHG3 in the proliferation, migration, and invasion of GC cell lines. The expressions of SNHG3, miR-326, and TWIST in GC9811-P GC cell lines were detected by RT-qPCR. Western blotting was performed to detect the protein levels of TWIST and EMT-related genes. Luciferase reporter gene analysis and RNA immunoprecipitation (RIP) analysis confirmed the interaction between lncRNA SNHG3, miR-326, and TWIST. CCK-8 and Transwell assays were performed to detect cell proliferation, invasion, and migration abilities. The results showed that lncRNA SNHG3 and TWIST were highly expressed in GC cell lines, while miR-326 was expressed to a low degree. Moreover, lncRNA SNHG3 knockdown or miR-326 overexpression significantly inhibited cell proliferation, migration, and invasion of GC cell lines. In addition, TWIST overexpression can reverse the inhibition of lncRNA SNHG3 knockdown or miR-326 overexpression on cell proliferation, migration, and invasion. In conclusion, lncRNA SNHG3 may promote GC progression through the miR-326/TWIST axis, which may provide a new diagnostic and prognostic biomarker for GC.

## 1. Introduction

Gastric cancer (GC) is a common malignancy in the world [1]. The most common cause of GC is *Helicobacter pylori* infection, accounting for more than half of the incidence. Other recognized risk factors include smoking, the intake of pickled vegetables in the diet, and the clinical management of obesity [2].The treatment of GC mainly includes surgery, chemotherapy, radiotherapy, and targeted therapy [3]. In the global scope, the overall results of GC are relatively poor, with a five-year survival of being lower than 10% [4]. Based on a comprehensive understanding of the biological basis of the disease, there is still an urgent need for accurate medicine. Long noncoding RNA (lncRNA) is more than 200 nucleotides in length without significant protein-coding potential [5]. It is worth noting that the accumulated evidence reveals that lncRNA plays an essential role in human malignant tumors [6]. SNHG3 is a new type of lncRNA, which may be related to Alzheimer's disease and colorectal cancer. For example, misaligned lncRNAs may act as oncogenes or tumor suppressor genes in the HCC process [7]. The results showed that the expression of Small Nucleolar RNA Host Gene 3 (SNHG3) was related to the malignant state, and the prognosis was relatively poor. SNHG3 was identified as a competitive endogenous RNA molecule to promote the malignant progression of colorectal cancer [8]. The abnormal upregulation of snhg3 in ovarian cancer is closely related to poor prognosis and malignant progression [9]. Systematic analysis of mitochondrial proteomics was performed [10]. LncRNA SNHG3 is also characterized by its involvement in the microRNA pathway of HCC, where miR-128/CD151 signaling of SNHG3 induces epithelial mesenchymal transition (EMT). Although SNHG3 has been recognized as a carcinogenic gene in a series of human cancers, the mechanism of SNHG3 in GC remains elusive.

In addition, we try to understand the basic molecular mechanism behind the carcinogenicity of lncRNA. Like lncRNA, miRNA has been widely recognized in cancer. MiR-326 plays an anticancer role in a variety of malignant tumors and targets different genes in glioma, endometrial cancer, and cervical cancer [11–14]. However, the interaction between SNHG3 and miR-326 in GC has not been reported. It has been reported that miR-326 associates with bone metastasis, a biochemical marker of bone turnover in lung cancer, and promotes EMT-induced cellular lung adenocarcinoma infiltration [15]. Wang et al. reported that miR-326 is abnormally expressed in metastasis and nonmetastasis tissues in non-small-cell lung cancer, providing an experimental basis for exploring the mechanism of non-small cell lung cancer metastasis and molecular diagnosis and treatment [16]. These results indicate that tumor suppresses the function of miR-326 in lung cancer. Our current goal is to investigate the biological function of miR-326 in GC and explore its potential mechanism.

TWIST, a transcription factor, plays an important role in the development and progression of cancer. The upregulation of TWIST inhibited the expression of E-cadherin in EMT, indicating that TWIST promoted metastasis by inducing EMT [17]. Previous investigations suggested that certain miRNA could regulate the expression level of TWIST through binding to TWIST mRNA [18, 19]. Previous reports have shown that miR-326 is dysfunctional and has various tumor developments. Nevertheless, the possible function and potential mechanism of miR-326 expression in GC have not been reported. In this study, we hypothesized that SNHG3 might promote HCC progression by targeting miR-326 expression. In order to clarify this problem, we analyzed the molecular expression mechanism of SNHG3 transcript in GC. The potential role of SNHG3 in tumor biology was studied.

## 2. Materials and Methods

### 2.1. Cell Culture and Transfection

Human immortalized gastric epithelial cell GES-1 and human gastric cancer cell lines HGC-27 and GC9811-P were cultured in DMEM medium containing 10% FBS (GIBCO, USA), 100 U/ml penicillin, and 100 *μ*g/ml streptomycin (Solarbio, China). Cells were cultured in a 5% CO_2_ at 37°C. For miRNA and siRNA transfection, HGC-27 and GC9811-P cells were laid in triplicate in a six-well plate. According to the manufacturer's procedures, miR-326 mimic or siRNA was transfected into GC cells using Lipofectamine 2000 (Invitrogen, USA).

### 2.2. CCK-8 Assay

According to manufacturer's instructions, cells were cultured in 96-well plates with a density of 2 × 10^3^ cells/well. The cells were cultured at 37°C for 24, 48, and 72 hours in an incubator containing 5% CO_2_. At different time points, 10 *μ*l CCK-8 solution was added to every pore and cultured at 37°C for another 2 hours. The absorbance at 450 nm was recorded.

### 2.3. Transwell Analysis

The determination was carried out by using a polycarbonate filter (8 *μ*m aperture). Twenty hours after miRNA transfection, cells were collected and 5 × 10^4^ cells in 200 *μ*l 0.1% serum medium were placed in the upper chamber. The lower chamber was full of 10% fetal bovine serum medium. After incubation for 24 hours and eliminating the cells in the upper chamber of the filter with cotton swabs, the cells below were fixed with 4% paraformaldehyde and stained with 0.1% crystal violet in 20% ethanol, and five phase contrast microscopes were randomly counted to observe the field of vision. Monitoring of migrating cells was performed by photographing with 400× magnification ICA microscope. The determination was carried out in triplicate.

### 2.4. Dual Luciferase Activity Assay

Shanghai gene Pharmaceutical Co. Ltd. (Shanghai, China) synthesized and confirmed the luciferase reporter vector pGL3-TWIST-3′UTR wild type (WT) and pGL3-TWIST-3′UTR mutant (MUT). GC cells were cotransfected with pGL3-TWIST-3′UTR WT or pGL3-TWIST-3′UTR MUT and miR-326 mimic or NC by Lipofectamine 2000. The transfected cells were harvested 48 hours after transfection, and luciferase activity was detected by a dual-luciferase reporter analysis system (Promega, Germany) according to the manufacturer's instructions.

### 2.5. RIP Assay

RIP analysis was performed using RIP Kit (Millipore, USA). HGC-27 and GC9811-P cell lines were lysed with RIP lysate buffer. The lysate was incubated overnight in RIP buffer with magnetic beads coupled to Ago2 or IgG antibodies (Sigma, USA) at 4°C. The expression of lncRNA SNHG3 and miR-326 was analyzed by RT-qPCR.

### 2.6. Quantitative Real-Time PCR

According to the manufacturer's instructions, total RNA was extracted from cultured cells using Trizol reagent (Invitrogen, USA). The RNA samples were then transcribed into cDNA in accordance with the manufacturer's instructions, with a total volume of 20 *μ*L. The same amount of cDNA sample was used as the template of RT-qPCR to detect the expression level of TWIST. RT-qPCR was carried out with a light cycle real-time PCR system and SYBR Green Master Mix (Takara). GAPDH was used as an endogenous reference, and each sample was standardized to its GAPDH content. All experiments were carried out in duplicate and repeated twice. 2^−ΔΔCt^ method was used to express the induction multiple.

### 2.7. Western Blot

Western blotting was performed on 10% twelve alkyl sulfate polyacrylamide gel electrophoresis (SDS-PAGE). The cells were transfected with miR-NC or miR-326.72 hours after transfection and were collected and lysed. The protein was separated and transferred to PVDF membrane by 10%SDS-PAGE gel. Then, 5% skim milk to block nonspecific connection was added. After incubation in dark for 1 hour at room temperature, rabbit anti-human TWIST polyclonal antibody was added in 1 : 200 dilutions. After washing 3 times with PBS, the membrane was mixed with the corresponding secondary antibody 1 hour at room temperature. For EMT-related proteins, the first antibody is used according to the specific protein. Finally, the strip was visualized by chemiluminescence after cleaning in TBS.

### 2.8. Statistical Analysis

Each experiment was repeated three times. SPSS 15.0 was used for statistical analysis. The Student–Newman–Keuls test is used as a posttest. *P* < 0.05 was supposed to indicate statistically significant differences.

## 3. Results

### 3.1. LncRNA SNHG3 and TWIST Were Upregulated while miR-326 Was Downregulated in HGC-27 and GC9811-P Cells

**Figure 1:**
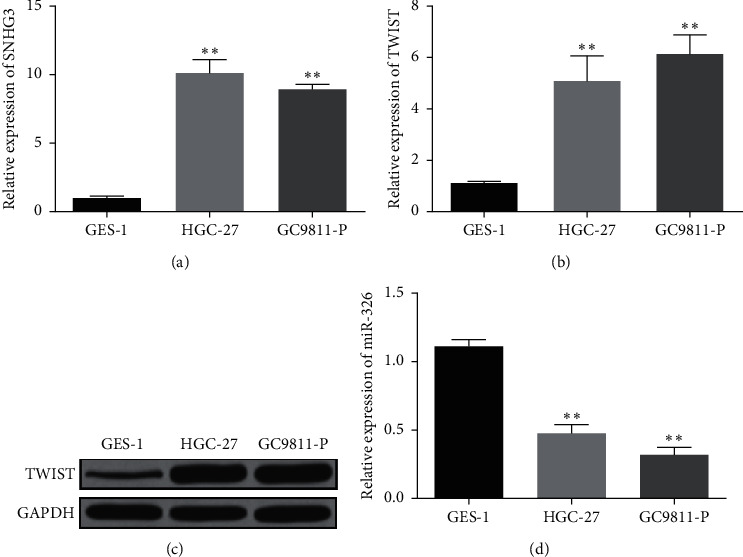
Expressions of lncRNA SNHG3, miR-326, and TWIST were examined in GC cells. (a) Expression of lncRNA SNHG3 in GC cells. (b) and (c) TWIST expression in GC cells. (d) MiR-326 expression in GC cells. ^*∗*^*P* < 0.05 and ^*∗∗*^*P* < 0.01.

Firstly, SNHG3, miR-326 and TWIST were detected in GES-1, HGC-27, and GC9811-P cell lines. The results showed that the expression of lncRNA SNHG3 and TWIST in HGC-27 and GC9811-P cell lines was higher than control group (Figures 1(a)–1(c)). However, the expression of miR-326 in HGC-27 and GC9811-P cell lines was obviously lower than that in GES-1 cell line (Figure 1(d)). These results indicated that the unusual expression of lncRNA SNHG3, miR-326, and TWIST might be related to the progress of GC.

### 3.2. LncRNA SNHG3 Knockdown or miR-326 Overexpression Suppressed Cell Proliferation, Migration, and Invasion in HGC-27 and GC9811-P Cells

**Figure 2:**
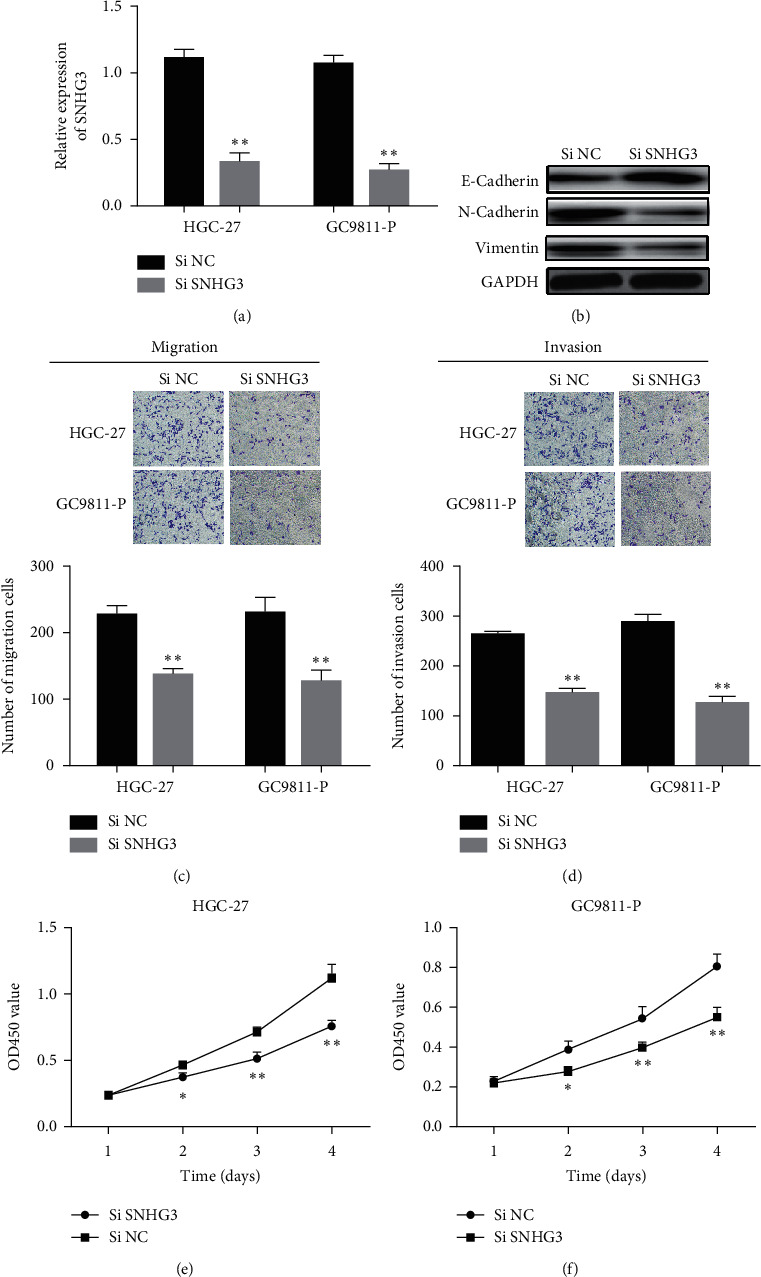
lncRNA SNHG3 knockdown suppressed HGC-27 and GC9811-P cell proliferation, migration, and invasion in vitro. (a) Expression of lncRNA SNHG3 in GC cells transfected with siSNHG3 or NC. (b) The protein level EMT-related marker in si NC and siSNHG3 groups. (c) and (d) Effect of lncRNA SNHG3 knockdown on cell migration and invasion. (e) and (f) Effect of lncRNA SNHG3 knockdown on cell proliferation. ^*∗*^*P* < 0.05 and ^*∗∗*^*P* < 0.01.

In order to study the functions of lncRNA SNHG3 and miR-326 in the development of GC, HGC-27 and GC9811-P cell lines were transfected with si SNHG3, miR-326 mimic, or their respective NC sequences, respectively. Compared with siNC, the expression of lncRNA SNHG3 was obviously decreased in cells transfected with siSNHG3 (Figure 2(a)). The influence of SNHG3 in protein levels of EMT-related proteins was detected by western blot. Results showed that the expressions of E-cadherin were significantly increased, while expressions of N-cadherin and vimentin were decreased (Figure 2(b)). In addition, cell migration and invasion were effectively reduced in HGC-27 and GC9811-P cell lines transfected with siSNHG3 (Figures 2(c) and 2(d)). CCK-8 assay showed that cell proliferation was significantly suppressed in cells transfected with siSNHG3 (Figures 2(e) and 2(f)).

**Figure 3:**
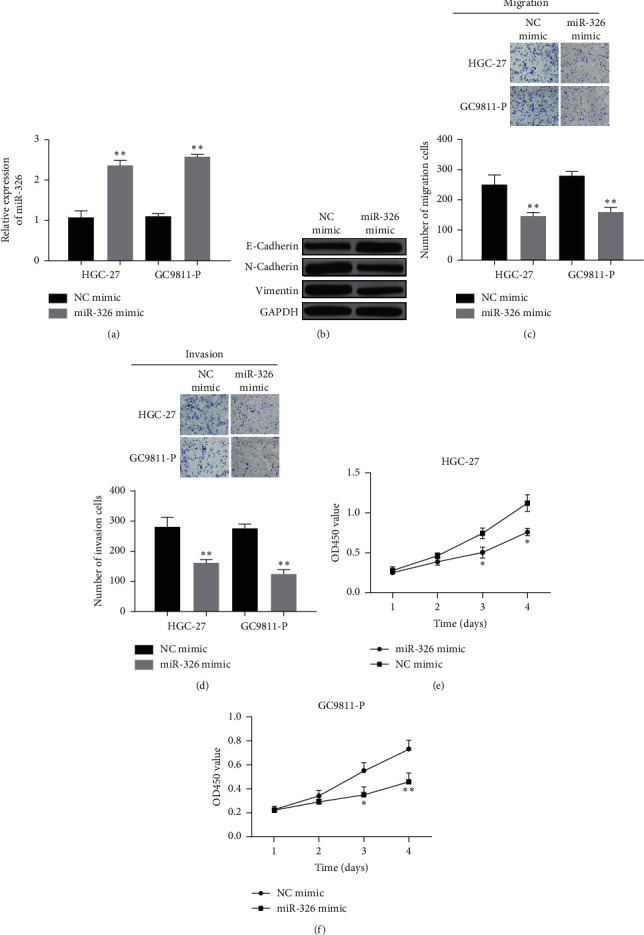
MiR-326 overexpression suppressed HGC-27 and GC9811-P cell proliferation, migration, and invasion in vitro. (a) Expression of miR-326 in GC cells transfected with miR-326 mimic or mimic NC. (b) The protein level expression of expression EMT-related marker in miR-326 mimic or mimic NC groups. (c) and (d) Effect of miR-326 overexpression on cell migration and invasion. (e) and (f) Effect of miR-326 overexpression on cell proliferation. ^*∗*^*P* < 0.05 and ^*∗∗*^*P* < 0.01.

miR-326 expression was obviously increased by miR-326 mimic in both HGC-27 and GC9811-P cell lines (Figure 3(a)). The protein level of EMT-related protein in cells transfected with miR-326 mimic was also detected (Figure 3(b)). Cell migration and invasion were effectively reduced by miR-326 mimic in HGC-27 and GC9811-P cell lines (Figures 3(c) and 3(d)). In the CCK-8 assay, cell proliferation of HGC-27 and GC9811-P cell lines was significantly suppressed by miR-326 mimic (Figures 3(e) and 3(f)).

### 3.3. LncRNA SNHG3 Targeted miR-326 and Negatively Regulated Its Expression

**Figure 4:**
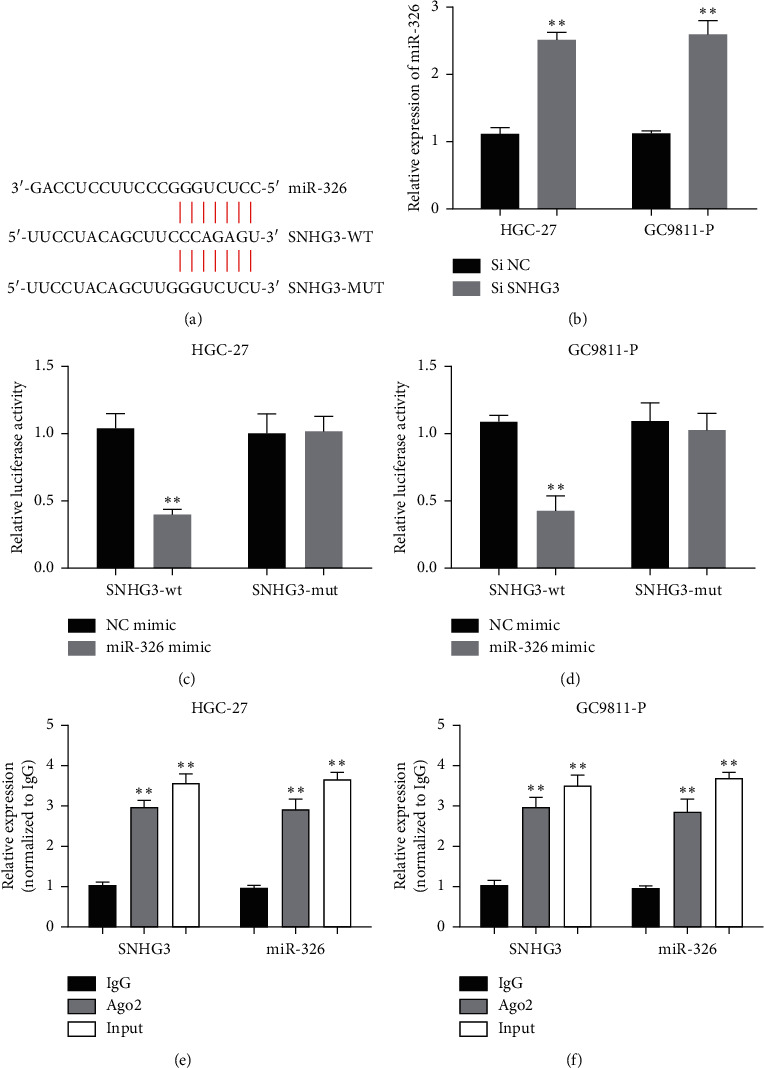
lncRNA SNHG3 functioned as an endogenous sponge to downregulate miR-326 by competitively binding to miR-326. (a) Putative binding sites between lncRNA SNHG3 and miR-326. (b) MiR-326 expression in GC cells transfected with siSNHG3 or siNC in HGC-27 and GC9811-P cell lines. (c) and (d) The luciferase activity was measured by a dual-luciferase reporter assay. (e) and (f) MiR-326 and lncRNA SNHG3 expressions in RIP assay. ^*∗∗*^*P* < 0.01.

It is confirmed that lncRNA, which can act as ceRNA, can bind to miRNA and release target RNA transcripts. In order to explore the regulatory mechanism of lncRNA SNHG3, starstarv3.0 predicted the target sites between lncRNA SNHG3 and miR-326 (Figure 4(a)). The expression level of miR-326 in HGC-27 and GC9811-P cell lines was investigated by RT-qPCR when the lncRNA SNHG3 was knocked down. Our study showed that the expression of miR-326 in HGC-27 and GC9811-P cell lines transfected with siSNHG3 increased significantly (Figure 4(b)). Therefore, we proposed that lncRNA SNHG3 could be used as ceRNA of miR-326 in GC cells. In addition, we used luciferase reporter gene assay to confirm the direct interaction between lncRNA SNHG3 and miR-326. After cotransfection of fluorescent enzyme report vector (SNHG3-wt or SNHG3-mut) and miR-326 mimic or NC into the cell line, we found that in HGC-27 and GC9811-P cell lines, the activity of fluorescent enzyme transfected with SNHG3-wt and miR-326 mimic decreased significantly (Figures 4(c) and 4(d)). In addition, to further verify the interaction between lncRNA SNHG3 and miR-326, RIP analysis was carried out with antibodies against Ago2. We found that both SNHG3 and miR-326 were rich in Ago2 precipitates in HGC-27 and GC9811-P cell extracts (Figures 4(e) and 4(f)). In general, these results suggested that lncRNA SNHG3 can be used as a ceRNA to bind directly to miR-326 and negatively regulate its expression.

### 3.4. TWIST Was a Target of miR-326 and Promoted by lncRNA SNHG3

**Figure 5:**
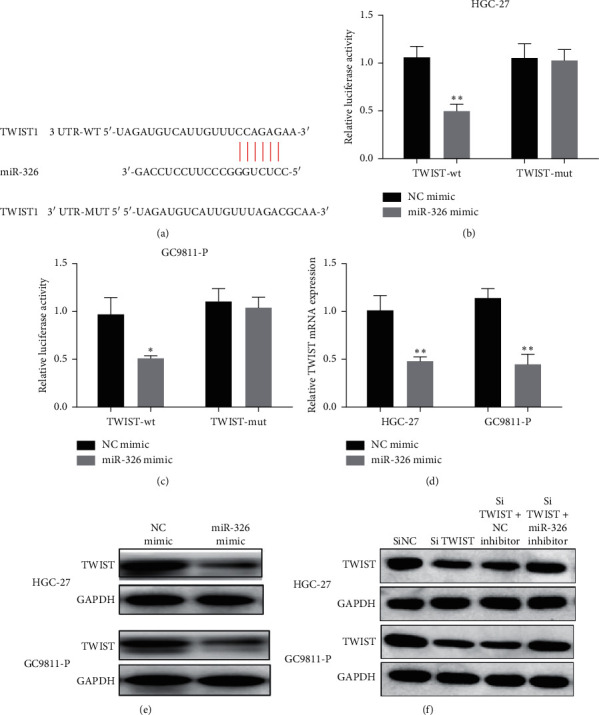
lncRNA SNHG3 regulated TWIST expression by competitively binding to miR-326. (a) Putative binding sites between miR-326 and TWIST. (b) and (c) The luciferase activity was measured by a dual-luciferase reporter assay. (d) and (e) TWIST expression in cells transfected with miR-326 mimic or NC. (f) The protein level of TWIST in HGC-27 and GC9811-P cells with different transfections.  ^*∗*^*P* < 0.05 and  ^*∗∗*^*P* < 0.01.

To further detect the molecular regulatory mechanism of lncRNA SNHG3 and miR-326, bioinformatics analysis was carried out to explore the potential target of miR-326 in GC cells. TWIST was predicted to be the main target gene of miR-326, and the target site was predicted by TargetScan (Figure 5(a)). In order to confirm the prediction of bioinformatics, we carried out luciferase reporter gene detection. Luciferase reporter vector (TWIST-wt or TWIST-mut) and miR-326 mimic or NC were cotransfected into HGC-27 and GC9811-P cells. Compared with the cotransfection of NC and TWIST-wt, the luciferase activity of cells cotransfected TWIST-wt and miR-326 mimic decreased significantly (Figures 5(b) and 5(c)). Furthermore, when the cells were transfected with miR-326 or NC, the expression of TWIST was measured. High expression of miR-326 effectively inhibited TWIST levels in HGC-27 and GC9811-P cells (Figures 5(d) and 5(e)). These data confirmed that TWIST was the target gene of miR-326 and was negatively controlled by miR-326. In addition, we found that cotransfection of miR-326 inhibitor and siSNHG3 could reverse the inhibition effects of siSNHG3 on TWIST expression (Figure 5(f)).

### 3.5. LncRNA SNHG3/miR-326/TWIST Axis Regulated Cell Proliferation, Migration, and Invasion in HGC-27 and GC9811-P Cells

**Figure 6:**
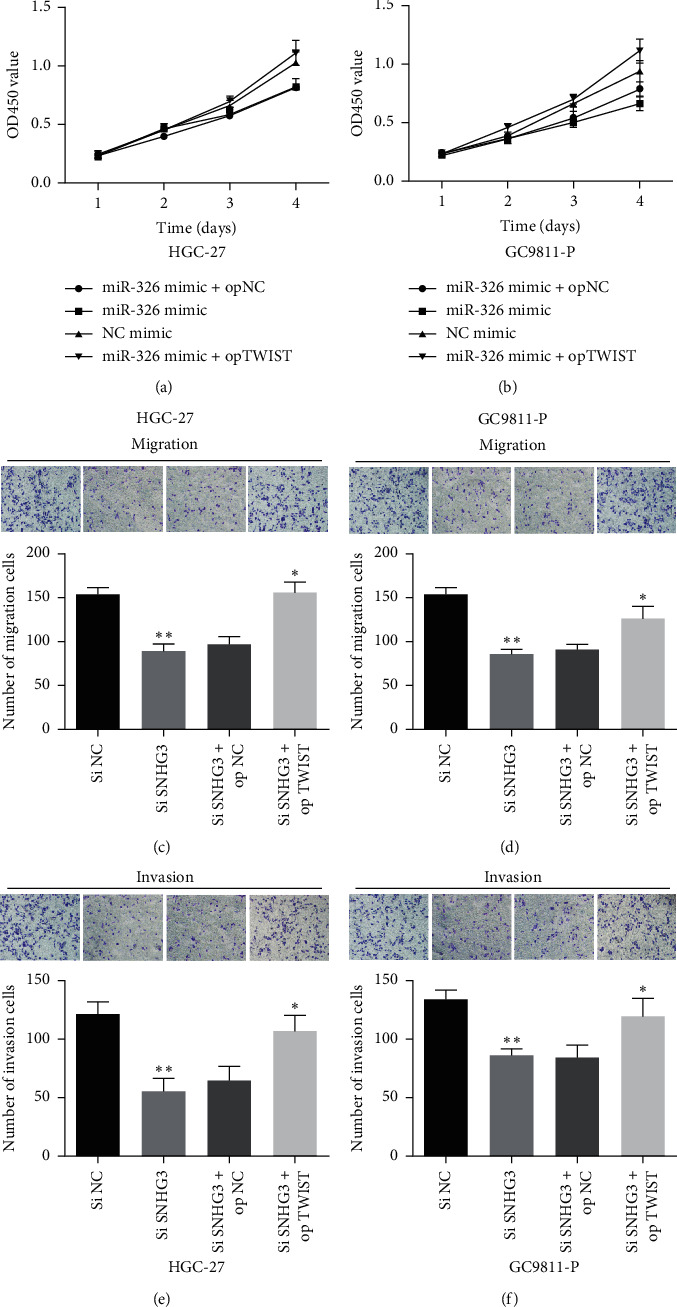
TWIST overexpression overturned lncRNA SNHG3 knockdown or miR-326 overexpression induced suppressive role on cell proliferation, migration, and invasion of GC cells. (a) and (b) TWIST overexpression overturned the suppressive functions of miR-326 overexpression in GC cell proliferation. ((c)–(f)) TWIST overexpression overturned the suppressive functions of SNHG3 knockdown in GC cell migration and invasion. ^*∗*^*P* < 0.05 and ^*∗∗*^*P* < 0.01.

According to the above experimental data, we speculated that the regulatory axis of lncRNA SNHG3-miR-326-TWIST might be related to the development of GC. As shown in Figures 6(a) and 6(b), the results showed that miR-326 overexpression significantly inhibited HGC-27 and GC9811-P cell proliferation, which could be partially reversed by TWIST overexpression. Furthermore, the inhibitory functions of si SNHG3 in HGC-27 and GC9811-P cell migration and invasion were also reversed by TWIST overexpression (Figures 6(c)–6(f)). It is suggested that lncRNA SNHG3 can promote cell proliferation, migration, and invasion by competitively binding and inhibiting miR-326 expression, thereby upregulating TWIST.

## 4. Discussion

GC is a major health problem and the second leading cause of cancer-related deaths worldwide. In Asia, about 60% of GC cases are diagnosed [20]. Despite improvements in surgical techniques and adjuvant therapies, GC is still highly lethal, with a 5-year survival rate of only 42% in China [21]. LncRNAs can mediate gene expression and affect tumor development, progression, and treatment. Huang et al. Snhg3 was identified as a competitive endogenous RNA molecule, which could promote the progression of colorectal cancer [22]. Hong et al. abnormal upregulation of snhg3 is closely related to poor prognosis in ovarian cancer [9]. However, many specific regulatory mechanisms of lncRNA in GC have not been studied. Therefore, exploring the potential molecular mechanism of lncRNA in GC may be helpful in the development of new diagnostic and therapeutic goals. Our study found that, according to RT-q PCR analysis, lncRNA SNHG3 was upregulated in GC cells compared to normal human immortalized gastric epithelial cells. CCK-8 and Transwell analysis showed that the inhibition of lncRNA SNHG3 could inhibit the proliferation, migration, and invasion of GC cells, which confirmed that lncRNA SNHG3 might affect the process of GC. Our results indicate that lncRNA SNHG3, which may be an oncogene, can be upregulated to benefit the development and progress of GC. However, the specific mechanism of lncRNA SNHG3 in GC needs further study.

Like ceRNA, lncRNA plays a key role in cancer biology and pathology by competitively inhibiting miRNA and indirectly regulating mRNA. Wu et al. results suggest that SNHG15 is a competitive endogenous RNA (ceRNA) that participated in regulating the YAP1-hippo signaling pathway in thyroid papillary carcinoma through miR-200a-3p [23]. NORAD plays an important role in regulating the function of osteosarcoma cells by competing with hsa-miR-199a-3p [24]. He et al. demonstrated that NORAD overexpression promotes the growth, invasion, and migration of PTC cells by inhibiting miR-202-5p expression [25]. LncRNA HULC accelerates the proliferation of colon cancer cells by miR-613/RTKN 28, while lncRNA Xist, as the endogenous sponge of miR-137, upregulates Rac1, which is the target gene of miR-137 and promotes the proliferation of glioma cells [26]. In our experiment, we found the expression of lncRNA SNHG3 and miR-326 and found that they showed a reverse expression trend in GC cells. In addition, according to bioinformatics analysis, we revealed that lncRNA SNHG3 contains a binding site with miR-326. Luciferase reporter gene and rip analysis showed that lncRNA SNHG3 interacted directly with miR-326 and regulated it. These results indicate that lncRNA SNHG3 acts as an endogenous sponge, competitively binds to miR-326, and negatively regulates its expression.

TWIST, a highly conserved transcription factor with a helical structure, is thought to regulate the carcinogenic properties essential for many cancer cells [27–29]. Recently, it was found that TWIST is involved in the transformation of breast cancer to stroma in vivo, which promotes epithelial formation [30]. It has also been reported that TWIST expression significantly enhances different types of cancer, such as prostate cancer, sarcoma, lymphoma, and melanoma [31–33]. In addition, TWIST also plays a significant role in many physiological processes, such as angiogenesis, visceral foot, exosmosis, and chromosome instability [31]. These findings indicate that TWIST is a new oncogene related to tumorigenesis and the progress of tumor. In order to investigate whether lncRNA SNHG3 and ceRNA can negatively regulate the release of miR-326, the upregulated target gene TWIST of miR-326 in GC cells was studied. Through bioinformatics analysis and luciferase reporter gene analysis, TWIST was proved to be the target of miR-326. More importantly, the overexpression of lncRNA SNHG3 reversed the luciferase activity of TWIST-wt inhibited by miR-326 mimic. Knockdown of lncRNA SNHG3 significantly reduced the expression of TWIST, which was reversed by miR-326 inhibitor. We suggest that lncRNA SNHG3 can regulate the expression of TWIST by negatively regulating miR-326. In addition, the overexpression of TWIST significantly overturned the inhibition of cell metastasis by knockdown of lncRNA SNHG3 or overexpression of miR-326, suggesting that the axis of lncRNA SNHG3-miR-326-TWIST may have participated in the development of GC.

## 5. Conclusion

In summary, our data showed that lncRNA SNHG3 promoted GC cell metastasis by regulating miR-326 and TWIST. This will help us to better understand the lncRNA, miRNA, and mRNA network in GC. However, the function of lncRNA SNHG3, miR-326, and TWIST was not tested in vivo. Moreover, we did not measure the expressions of SNHG3 in clinical patients. Further research should also be carried out to explore whether certain signaling pathways play a role in the mechanism of lncRNA SNHG3 in GC. Thus, the significance and robustness of SNHG3 as a biomarker for GC requires further confirmation.

## Data Availability

All primary data are available from the corresponding author upon reasonable request.
