# Corrigendum to “LncRNA SNHG3 Promotes Gastric Cancer Cells Proliferation, Migration, and Invasion by Targeting miR-326”

**DOI:** 10.1155/2022/9780315

**Published:** 2022-09-20

**Authors:** Jun Rao, Jinjin Fu, Chuchen Meng, Jin Huang, Xiangrong Qin, Shaohua Zhuang

**Affiliations:** ^1^Department of Gastroenterology, Changzhou No. 2 People's Hospital, The Affiliated Hospital of Nanjing Medical University, No. 29 Xinglong District, Changzhou 213000, China; ^2^Department of Endocrinology, Changzhou No. 2 People's Hospital, The Affiliated Hospital of Nanjing Medical University, Changzhou, China

In the article titled “LncRNA SNHG3 Promotes Gastric Cancer Cells Proliferation, Migration, and Invasion by Targeting miR-326” [1], concerns with the figures have been identified as initially raised on PubPeer [2].

In the published article, the right side panel of Figure 2(c) is the same as the middle two panels of Figure 6(c), the first left panel of Figure 2(d) is the same as the first left panel of Figure 3(d), and the N-cadherin of Figure 2(b) is the same as TWIST in HGC-27 of Figure 5(e). The authors explained that these errors were introduced during the preparation of the manuscript, and this does not affect the results and conclusions of the article.

While the authors initially responded to provide an explanation to the concerns along with the revised figures, they have not responded to requests to approve this notice. This corrigendum is therefore published with the agreement of the editorial board to ensure the appropriate correction of the issues detailed above. The corrected Figures 2 and 6 are as follows.

## Figures and Tables

**Figure 1:**
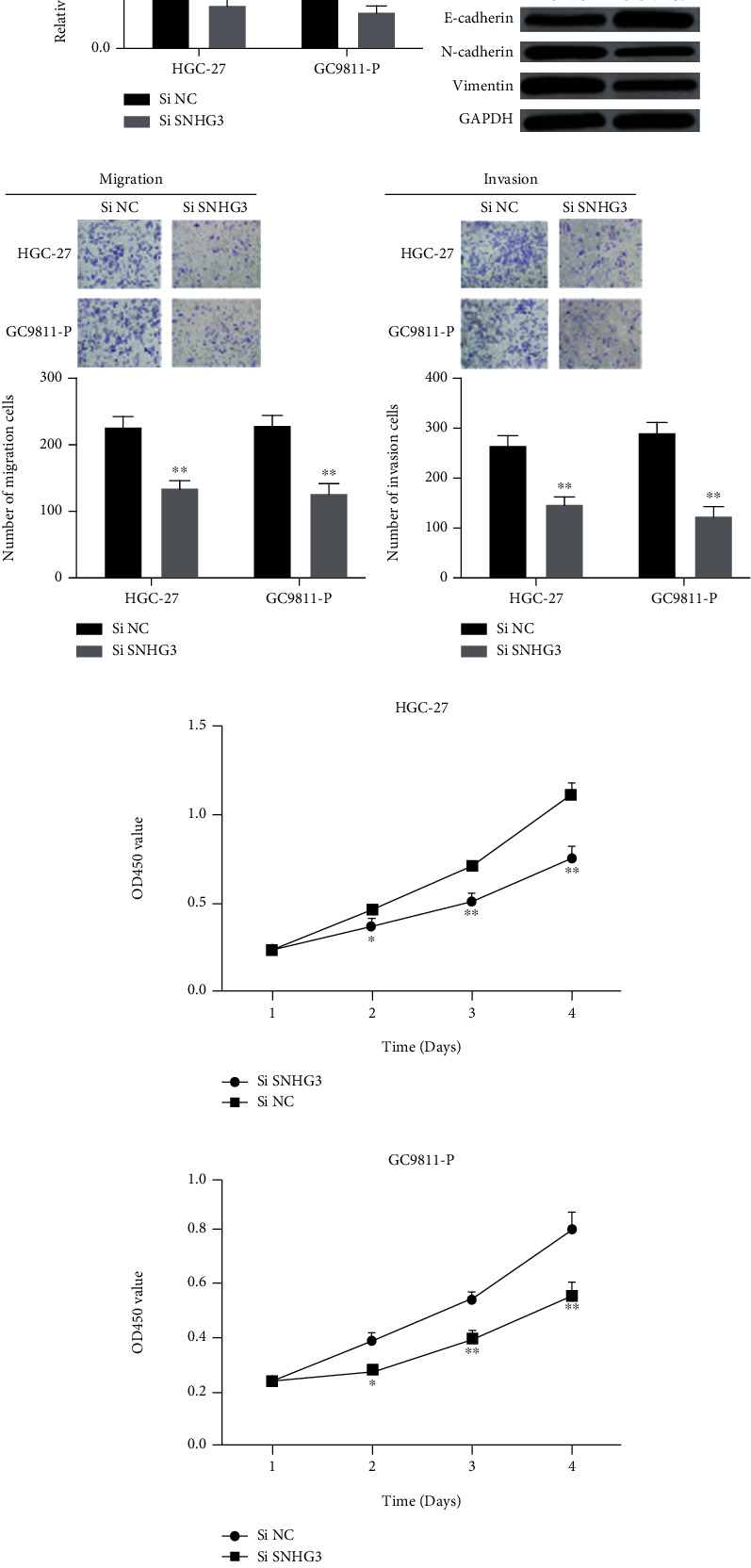
lncRNA SNHG3 knockdown suppressed HGC-27 and GC9811-P cell proliferation, migration, and invasion in vitro. (a) Expression of lncRNA SNHG3 in GC cells transfected with siSNHG3 or NC. (b) The protein level EMT-related marker in si NC and siSNHG3 groups. (c, d) Effect of lncRNA SNHG3 knockdown on cell migration and invasion. (e, f) Effect of lncRNA SNHG3 knockdown on cell proliferation. ^∗^*P* < 0.05 and ^∗∗^*P* < 0.01.

**Figure 2:**
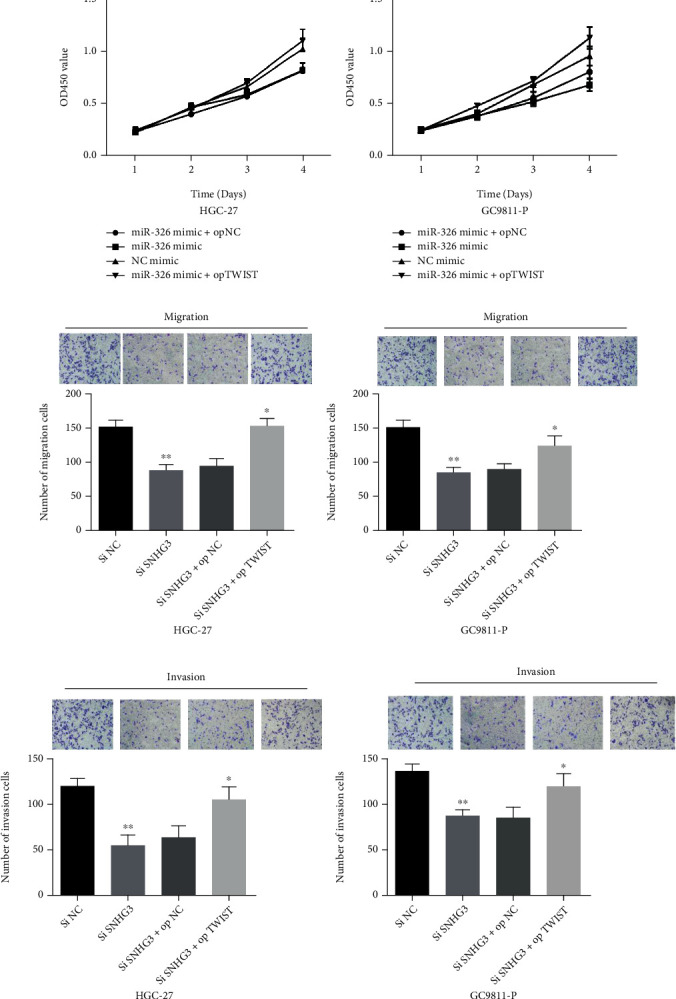
TWIST overexpression overturned lncRNA SNHG3 knockdown or miR-326 overexpression induced suppressive role on cell proliferation, migration, and invasion of GC cells. (a, b) TWIST overexpression overturned the suppressive functions of miR-326 overexpression in GC cell proliferation. (c–f) TWIST overexpression overturned the suppressive functions of SNHG3 knockdown in GC cell migration and invasion. ^∗^*P* < 0.05 and ^∗∗^*P* < 0.01.
